# Nitric oxide and oxidative stress pathways do not contribute to sex differences in renal injury and function in Dahl SS/Jr rats

**DOI:** 10.14814/phy2.14440

**Published:** 2020-07-11

**Authors:** Hannah R. Turbeville, Ashley C. Johnson, Michael R. Garrett, Elena L. Dent, Jennifer M. Sasser

**Affiliations:** ^1^ Department of Pharmacology and Toxicology University of Mississippi Medical Center Jackson MS USA; ^2^ Department Physiology and Biophysics University of Mississippi Medical Center Jackson MS USA

**Keywords:** Dahl S, kidney disease, nitric oxide, Oxidative stress, renal fibrosis, renal function, salt‐sensitive hypertension

## Abstract

The burden of hypertension in the United States is increasing and yields significant morbidity and mortality, and sex differences in hypertension are widely recognized. Reduced nitric oxide (NO) bioavailability and increased oxidative stress are known to contribute to the pathogenesis of hypertensive renal injury, and but their contributions to sex differences in injury progression of are undefined. Our purpose was to test the hypothesis that male hypertensive rats have accelerated renal injury compared to females and to determine the contributions of the nitric oxide pathway and oxidative stress in these differences. Male and female Dahl SS/Jr rats, a model that spontaneously develops hypertension with age, were allowed to age on a 0.3% NaCl diet until 3 or 6 months of age, at which points blood pressure was measured and plasma, tissue, and urine were collected. While no significant sex differences in blood pressure were present at either time point, renal injury measured by urine protein excretion was more severe (male = 44.9 ± 6; female = 15±3 mg/day/100 g bw, *p* = .0001), and renal function was reduced (male = 0.48 ± 0.02; female = 0.7 ± 0.03 ml min^‐1^ g^‐1^ kw, *p* = .001) in males compared to females with age. Both male and female rats exhibited reduced nitric oxide metabolites (3 months: male = 0.65 ± 0.1; female = 0.74 ± 0.3; 6 months: male = 0.16 ± 0.1; female = 0.41 ± 0.1 ml min^‐1^ g^‐1^ kw, p, age = 0.02, p, sex = 0.3). Levels of urinary TBARS were similar (3 months: male = 20±1.5; female = 23±1.8; 6 months: male = 26±4.8; female = 23±4.7µM day g^‐1^ kw, *p*, age = 0.4, *p*, sex = 0.9), extracellular superoxide dismutase (EC SOD) mRNA was greater in females (3 months: male = 0.35 ± 0.03; female = 1.4 ± 0.2; 6 months: male = 0.4 ± 0.05; female = 1.3 ± 0.1 normalized counts, *p*, age = 0.7, *p*, sex < 0.0001), but EC SOD protein expression was not different (3 months: male = 0.01 ± 0.002; female = 0.01 ± 0.002; 6 months: male = 0.02 ± 0.004; female = 0.01 ± 0.002 relative density, *p*, age = 0.2, *p*, sex = 0.8). These data support the presence of significant sex differences in renal injury and function in the Dahl S rat and identify a need for further study into the mechanisms involved.

## Graphical abstract

Reduced nitric oxide bioavailability and increased oxidative stress are known to contribute to the pathogenesis of hypertensive renal injury, and but their contributions to sex differences in injury progression of are undefined. Our purpose was to test the hypothesis that male hypertensive rats have accelerated renal injury compared to females and to determine the contributions of the nitric oxide pathway and oxidative stress in these differences. Our data support the presence of significant sex differences in renal injury and function in the Dahl S rat and identify a need for further study into the mechanisms involved.

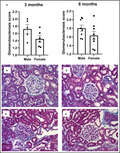

## 1 INTRODUCTION

Hypertension is a major contributor to morbidity and mortality worldwide. In the United States, age‐matched prevalence of hypertension from 2015 to 2016 was 46% of the population, and the absolute burden of hypertension has increased (Dorans, Mills, Liu, & He, 2018; Virani et al., 2020). Data from the National Health and Nutrition Examination Survey (NHANES) from 2009 to 2012 indicate that only slightly over half (54.1%) of all those with hypertension were “controlled,” or adequately managed on their current medical therapy by the most recent blood pressure (BP) guidelines. The American Heart Association 2020 Update reports that the death rate attributable to hypertension rose 25.7% in the decade from 2007 to 2017 and the number of actual deaths increased by 56.1% (Virani et al., 2020). The pathogenesis of hypertension is multifactorial, and likely differs on a patient‐to‐patient basis (Oparil, Zaman, & Calhoun, 2003).

Epidemiologic data show sex differences in the prevalence of hypertension. Before 45 years of age, there is a higher prevalence of hypertension men than women, whereas after 65 years of age, prevalence increases in women. From 45 to 64 years of age, there is no difference in prevalence between the sexes (Mozaffarian et al., 2016; Virani et al., 2020). However, these differences are not reflected in current guidelines for BP management, likely due to a lack of evidence regarding etiology, progression, and treatment relevant to sex‐specific hypertension, while specific recommendations based on age and race do appear (Whelton et al., 2018). While subgroup analysis from the SPRINT trial demonstrated no significant difference in outcomes for men and women (Foy et al., 2018), this is likely due to the gross underrepresentation of women compared to men in this trial (36% women, 64% men), which is a common and persistent issue in many clinical trials. Additional rationale for the discrepancy can be found in the analysis by Delles and Currie (2018).

Sex differences are also observed in several animal models of hypertension, including the angiotensin II‐infusion model in mice and rats, the Dahl S rat, and the L‐NAME rat infusion model (Hinojosa‐Laborde, Lange, & Haywood, 2000; Ouchi, Share, Crofton, Iitake, & Brooks, 1987; Pollow et al., 2014; Sainz et al., 2004; Xue et al., 2009; Xue, Pamidimukkala, & Hay, 2005). While many studies focus on the contributions of chromosomal factors and gonadal hormones to these differences (Ji et al., 2010; Sainz et al., 2004; Xue et al., 2009), additional mechanisms are also being studied, such as differences in immune cell infiltration (Gillis & Sullivan, 2016; Pollow et al., 2014). Nevertheless, studies directly examining the molecular mechanisms underlying sex differences in hypertension are needed to further elucidate both the etiology and potential therapeutic targets.

Oxidative stress produced by inflammation and immune cell infiltration is a known contributor to both hypertension and kidney disease (Meng, Nikolic‐Paterson, & Lan, 2014; Rodriguez‐Iturbe, Pons, & Johnson, 2017). Specifically, it seems that the interrelated mechanisms of inflammation, immune cell infiltration and activation, oxidative stress, and remodeling contribute to a self‐perpetuating cycle of damage (Vaziri & Rodriguez‐Iturbe, 2006). Immune cells are a source of proinflammatory cytokines and profibrotic chemokines that lead to extracellular remodeling and collagen deposition, and oxidative stress acts as a chemoattractant for immune cells in addition to causing direct cellular damage. Oxidative stress is shown to increase renal vascular resistance (RVR), which is improved by addition of an antioxidant superoxide dismutase (SOD) mimetic (Schnackenberg, Welch, & Wilcox, 1998; Zou, Li, & Cowley, 2001). Improvement in RVR seems to be mediated through the nitric oxide (NO) pathway, since inhibition of NO production with L‐NAME prevents the antihypertensive effect of the SOD mimetic (Schnackenberg et al., 1998). The changes in RVR in response to oxidative stress also exert an influence on the nearby endothelial cell phenotypes, inducing cellular damage, changes in gene expression transcription, and adhesion molecule expression, ultimately leading to microvascular dysfunction (Li & Shah, 2004; True, Rahman, & Malik, 2000).

Human studies and animal models of hypertension have demonstrated increased oxidative stress and decreased antioxidant mechanisms (Beswick, Dorrance, Leite, & Webb, 2001; Grunfeld et al., 1995; Kumar & Das, 1993; Meng, Cason, Gannon, Racusen, & Manning, 2003; Pedro‐Botet, Covas, Martin, & Rubies‐Prat, 2000; Russo et al., 1998; Schnackenberg et al., 1998). While levels of reactive oxygen species (ROS) have varied in human studies, several studies found consistent decreases in the antioxidant enzyme SOD in hypertensive patients compared to controls (Kumar and Das, (1993); Pedro‐Botet et al., 2000; Russo et al., 1998). In addition to direct cellular damage, ROS contributes to disease and damage through secondary mechanisms such as inactivation of NO (Beckman & Koppenol, 1996). Decreases in NO have been noted in hypertensive patients with a concurrent increase in ROS, which were returned to normal after achieving BP control (Kumar & Das, 1993). Furthermore, low levels of NO have been shown to act in a signaling capacity, increasing expression of antioxidants (Keller et al., 2003), and protecting against oxidative damage and fibrosis (Dreieicher et al., 2009; Pleskova et al., 2006; Schaefer et al., 2003). Upregulation or maintenance of endothelial NO synthase (NOS3) has been suggested as one mechanism for renal protection produced by the statin and β‐blocker drug classes (Kobayashi et al., 2008; Mason et al., 2006; Zhou, Jaimes, & Raij, 2004; Zhou, Schuman, Jaimes, & Raij, 2008). While clinical trials with nonspecific antioxidant therapies have largely failed to show benefits in hypertension, more specifically targeted therapies may show promise if the contributory mechanism can be identified. For example, if oxidative stress is found to significantly contribute to the etiology of hypertension in a single subgroup, such as a single sex, these therapies can be targeted to the group that will achieve the highest response rate.

Oxidative stress has also been shown to contribute to the pathogenesis of CKD in humans. Markers of oxidative stress have been shown to increase significantly with severity of CKD and are reduced by hemodialysis (Dounousi et al., 2006; Karamouzis et al., 2008). Yilmaz et al. (2006) showed higher levels of markers of oxidative stress and lower levels of antioxidants in patients with CKD as compared to controls, as well as a negative correlation between markers of oxidative stress and glomerular filtration rate (GFR). Furthermore, the expression of extracellular superoxide dismutase (EC SOD), a key antioxidant enzyme, is significantly decreased in renal samples of patients with fibrotic proteinuric CKD (Tan et al., 2015). Meta‐analysis of clinical trials using antioxidants in patients on dialysis or with CKD demonstrated potential for improvement of creatinine clearance and reduced development of end‐stage kidney disease, though all studies involved were relatively small (Jun et al., 2012).

The Dahl SS/Jr rat is an established model of salt‐sensitive hypertension that develops hypertension and renal injury with age in the absence of high salt. The progression of hypertension and renal injury in this strain on a low salt (0.3% NaCl) diet provides an avenue for the study of age‐related disease progression without the excess mortality present on a high salt diet (Cicila et al., 1997, 2009; Garrett, Dene, & Rapp, 2003). The use of salt‐sensitive animals is also reflective of the affected population, since a majority of individuals with hypertension are known to be at least somewhat salt‐sensitive (Elijovich et al., 2016). The purpose of this study was to test the hypothesis that male Dahl S hypertensive rats have accelerated renal injury and dysfunction compared to females and to determine the contributions of the nitric oxide pathway and oxidative stress to these differences.

## 2 METHODS

### 2.1 Animals

Dahl salt‐sensitive (SS/Jr) rats were obtained from the colony maintained by Dr. Michael Garrett at the University of Mississippi Medical Center. All rats were fed normal chow (TD7034, 0.3% NaCl, Harlan Teklad, Madison, WI) and water ad libitum on a 12‐hr light/dark cycle. Development of hypertension in Dahl SS/Jr rats on a 0.3% NaCl diet has been previously established, and higher content salt diets act only to hasten the onset and increase mortality (Cicila et al., 1997, 2009; Garrett et al., 2003; Rapp & Dene, 1985). Male and female rats were mated in nonconsanguineous groups, and the presence of sperm in a vaginal swab was indicative of gestational day one. Lactating dams and pups were on normal chow for the duration of the study, and pups were weaned at 4 weeks of age. Measurements were made and tissues collected on separate groups of offspring at 3 and 6 months of age. One male and one female pup per litter were maintained to each time point with up to three same‐sex cagemates. Animals were assessed regularly by laboratory and animal facility staff for signs of illness, cachexia, or other health concerns and were removed from the studies if necessary. All experiments were performed in accordance with the National Institutes of Health Guide for the Care and Use of Laboratory Animals and were monitored by the University of Mississippi Medical Center Institutional Animal Care and Use Committee.

### 2.2 Systolic blood pressure measurements

Systolic BP measurements were obtained using the volumetric pressure recording tail cuff method (CODA 8‐channel system, Kent Scientific Corp., Torrington, CT). Animals were trained in restraints alone followed by training in restraints on the heated platform before obtaining sets of no less than five valid measurements each on two consecutive days. Training and measurements took place in the same room adjacent to animal housing before noon on each respective day. Measurements for each animal were averaged to obtain final BP measurement.

### 2.3 Urinary Measurements

Rats were placed in metabolic cages adjacent to their original housing for 24‐hr urine collection prior to tissue harvest. Urinary protein excretion was determined by Bradford Assay (Bio‐Rad Laboratories). Urinary excretion rates of KIM‐1 (R&D Systems, Minneapolis, MN) and nephrin (ABclonal, Woburn, MA) were quantified via commercially available ELISA assays. Urinary nitrate/nitrite excretion (NOx) was measured using a colorimetric assay (Cayman Chemical, Ann Arbor, MI). Urinary thiobarbituric acid reactive substances (TBARS) were measured via a chemical assay (R&D Systems, Minneapolis, MN).

### 2.4 Tissue collection

When rats reached either 3 or 6 months of age, subgroups were anesthetized using inhaled isoflurane anesthesia (5% for induction, 2%–3% for maintenance, Piramal Healthcare). A terminal blood sample was obtained from the abdominal aorta into heparinized syringes, and the organs were subsequently perfused blood‐free with saline. Euthanasia was performed by excising the heart. The kidneys were removed, dissected into cortical and medullary regions, and snap frozen in liquid nitrogen for later analysis.

### 2.5 Renal histological analysis

A cross‐section of the left kidney of all rats was fixed in 10% formalin, paraffin embedded, cut into 4 µm sections, and stained with Masson's trichrome stain. Glomerular injury was assessed on a scale from 0 (normal) to 4 (severe) for 25 glomeruli per sample as previously described (Raij, Azar, & Keane, 1984).

### 2.6 Creatinine clearance measurements

Terminal blood samples were centrifuged, and plasma was isolated. Creatinine concentrations were measured in both urine and plasma samples (Vet Axcel Chemistry Analyzer, Alfa Wasserman, West Caldwell, NJ) and then used to calculate creatinine clearance for each animal.

### 2.7 Western blots

Renal cortices were isolated from right and left kidneys collected at euthanasia. Kidney sections were homogenized in lysis buffer (Tris 20 mM, EDTA 5 mM, EGTA 10 mM, sodium orthovanadate 1 mM, Triton X‐100 1%, sodium pyrophosphate 2.5 mM, B‐glycerophosphate 1 mM, DTT 2 mM, PMSF 0.1 mg/ml, leupeptin 0.01 mg/ml, aprotinin 0.01 mg/ml) using the Next Advance Bullet Blender. Total protein concentration for each sample was determined using a DC protein assay (Bio‐Rad Laboratories). Homogenized samples were standardized by their protein concentrations (200 μg per lane), separated by electrophoresis (140 V until fully separated, 4%–15% TGX Stain‐Free Precast gel, Bio‐Rad Laboratories), and then transferred to nitrocellulose membranes (Bio‐Rad Trans‐Blot Turbo). Gels and membranes were imaged (ChemiDoc MP Imaging System and Image Lab 3.0 Software, Bio‐Rad Laboratories) to determine transfer efficiency/uniformity and equal loading. Each membrane was incubated overnight with the primary antibody (rabbit anti‐Cu/Zn SOD [1:2000], Enzo Life Sciences SOD‐101; rabbit anti‐Mn SOD [1:2000], Enzo Life Sciences SOD‐111; rabbit anti‐EC SOD [1:1000], Enzo Life Sciences SOD‐106, antibodies previously validated by Ho et al. (2016)), at 4°C on a rocking plate. Membranes were then incubated with a fluorescent secondary antibody (goat anti‐rabbit [1:3000] for Cu/Zn and Mn SOD, [1:2000] for EC SOD, Bio‐Rad Laboratories StarBright Blue700) for 1 hr at room temperature. Bands were quantified by densitometry using the Image Lab 3.0 Software (Bio‐Rad Laboratories). After subtraction of background, protein abundance was calculated as integrated optical density of the protein of interest, factored for total protein loading for each sample.

### 2.8 Targeted RNA sequencing

Expression analysis was performed on genes involved in inflammation, glomerular function, renal injury, and reactive oxygen species. RNA was isolated from kidney using an automated KingFisher™ Flex nucleic acid system along with a KingFisher™ Pure RNA Kit. RNA was evaluated for quantity (Nanodrop One and Qubit Fluorimeter) and quality using Qiagen QIAxcel Advanced system. The Illumina DesignStudio application (http://designstudio.illumina.com/) was utilized to design custom amplicons across exon‐intron boundaries of target genes (*n* = 32 gene with 1–2 probes per gene). The gene target/probes that were designed/used are listed in Tables 1 and 2. Based on the DesignStudio output, the TruSeq Targeted RNA Custom Panel Kit was ordered and subsequently utilized to prepare a library for collected RNA samples. The Illumina MiSeq platform allows for analysis of pooled libraries [e.g., *n* = 96–384 RNA samples) to be processed at a single time as individual samples will have a unique “barcode.” Libraries were sequenced on Illumina MiSeq using MiSeq Reagent Kit v2 (150 cycle). Sequencing reads were demultiplexed and aligned to rn6 genome assembly using RNA Amplicon Application (along with custom panel manifest) available on Illumina BaseSpace Computing Platform (http://basespace.illumina.com/). Aligned reads for each gene were normalized to count per million for downstream analysis.

**Table 1:** Gene targets and corresponding proteins quantified by targeted RNA sequencing as described in methods

Gene Identifier	Protein
agtr1a	Angiotensin II Receptor, type 1A
CAT	Catalase
col3a1	Collagen Type III Alpha 1 chain
edn1	Endothelin−1
ednra	Endothelin Receptor type A
endrb	Endothelin Receptor type B
GPX2	Glutathione Peroxidase 2
GSS	Glutathione Synthetase
hmox1	Heme Oxygenase 1
hmox2	Heme Oxygenase 2
hif3a	Hypoxia‐Inducible Factor 3 Alpha
IL10	Interleukin 10
IL17A	Interleukin 17A
IL6	Interleukin 6
HAVCR1	Hepatitis A Virus Cellular Receptor 1
NOX4	NADPH Oxidase 4
NPHS1	Nephrin
LCN2	Lipocalin 2
NOs2	Nitric Oxide Synthase 2, inducible nitric oxide synthase
NOS3	Nitric Oxide Synthase 3, endothelial nitric oxide synthase
pde5a	Phosphodiesterase 5A
nphs2	Podocin
prkg2	cGMP‐dependent protein kinase G
atp6ap2	V‐type proton ATPase
s100a4	S100 calcium binding protein A4
sod1	Superoxide Dismutase 1
sod2	Superoxide Dismutase 2
sod3	Superoxide Dismutase 3
tgfb1	Tumor Growth Factor β1
timp1	TIMP metallopeptidase inhibitor 1
tnf	Tumor necrosis factor
vim	Vimentin

**Table 2:** Gene targets and probes used in targeted RNA sequencing as described in methods

Gene Name	Transcript ID	Left Exon	Right Exon	Species	Chromosome	Start	End
Agtr1a	NM_030985	1	0	*Rattus norvegicus*	chr17	40,684,884	40,675,481	
Agtr1a	NM_030985	2	1	*Rattus norvegicus*	chr17	40,675,458	40,631,259	
Cat	NM_012520	6	7	*Rattus norvegicus*	chr3	88,672,303	88,672,855	
Cat	NM_012520	9	10	*Rattus norvegicus*	chr3	88,680,122	88,683,020	
Col3a1	NM_032085	13	14	*Rattus norvegicus*	chr9	44,299,016	44,299,719	
Col3a1	NM_032085	49	50	*Rattus norvegicus*	chr9	44,316,672	44,317,443	
Edn1	NM_012548	2	1	*Rattus norvegicus*	chr17	28,307,715	28,306,343	
Edn1	NM_012548	4	3	*Rattus norvegicus*	chr17	28,305,981	28,304,545	
Ednra	NM_012550	2	3	*Rattus norvegicus*	chr19	32,081,838	32,095,813	
Ednra	NM_012550	6	7	*Rattus norvegicus*	chr19	32,103,022	32,105,026	
Ednrb	NM_017333	3	2	*Rattus norvegicus*	chr15	87,896,604	87,893,678	
Ednrb	NM_017333	2	1	*Rattus norvegicus*	chr15	87,897,524	87,896,651	
Gpx2	NM_183403	1	0	*Rattus norvegicus*	chr6	99,375,916	99,373,055	
Gss	NM_012962	5	4	*Rattus norvegicus*	chr3	146,072,936	146,072,201	
Gss	NM_012962	12	11	*Rattus norvegicus*	chr3	146,059,093	146,058,026	
Hmox1	NM_012580	2	3	*Rattus norvegicus*	chr19	13,966,589	13,968,156	
Hmox1	NM_012580	1	2	*Rattus norvegicus*	chr19	13,965,273	13,966,155	
Hmox2	NM_024387	2	1	*Rattus norvegicus*	chr10	10,918,090	10,917,095	
Hmox2	NM_024387	1	0	*Rattus norvegicus*	chr10	10,929,339	10,918,153	
Hif3a	NM_022528	14	13	*Rattus norvegicus*	chr1	77,379,020	77,377,608	
Hif3a	NM_022528	10	9	*Rattus norvegicus*	chr1	77,395,493	77,384,150	
Il10	NM_012854	3	4	*Rattus norvegicus*	chr13	43,956,466	43,957,558	
Il10	NM_012854	0	1	*Rattus norvegicus*	chr13	43,954,037	43,954,961	
Il17a	NM_001106897	0	1	*Rattus norvegicus*	chr9	19,455,037	19,456,177	
Il6	NM_012589	3	2	*Rattus norvegicus*	chr4	459,586	458,654	
Il6	NM_012589	4	3	*Rattus norvegicus*	chr4	458,568	457,278	
Havcr1	NM_173149	5	6	*Rattus norvegicus*	chr10	31,851,805	31,853,749	
Havcr1	NM_173149	3	4	*Rattus norvegicus*	chr10	31,844,938	31,848,575	
Nox4	NM_053524	16	17	*Rattus norvegicus*	chr1	143,590,456	143,603,070	
Nox4	NM_053524	11	12	*Rattus norvegicus*	chr1	143,506,962	143,564,685	
Nphs1	NM_022628	27	28	*Rattus norvegicus*	chr1	85,439,246	85,442,407	
Nphs1	NM_022628	19	20	*Rattus norvegicus*	chr1	85,427,263	85,430,136	
Lcn2	NM_130741	1	0	*Rattus norvegicus*	chr3	11,514,593	11,514,017	
Lcn2	NM_130741	2	1	*Rattus norvegicus*	chr3	11,513,935	11,512,859	
Nos2	NM_012611	25	26	*Rattus norvegicus*	chr10	65,070,187	65,072,223	
Nos2	NM_012611	8	9	*Rattus norvegicus*	chr10	65,052,926	65,054,621	
Nos3	NM_021838	13	12	*Rattus norvegicus*	chr4	6,170,899	6,166,281	
Nos3	NM_021838	23	22	*Rattus norvegicus*	chr4	6,160,552	6,159,872	
Pde5a	NM_133584	7	8	*Rattus norvegicus*	chr2	219,483,379	219,484,314	
Pde5a	NM_133584	20	21	*Rattus norvegicus*	chr2	219,547,581	219,550,380	
Nphs2	NM_130828	6	7	*Rattus norvegicus*	chr13	71,307,268	71,308,198	
Nphs2	NM_130828	3	4	*Rattus norvegicus*	chr13	71,302,117	71,303,654	
Prkg2	NM_013012	11	12	*Rattus norvegicus*	chr14	11,946,797	11,969,486	
Prkg2	NM_013012	17	18	*Rattus norvegicus*	chr14	12,002,461	12,004,842	
Atp6ap2	NM_001007091	3	2	*Rattus norvegicus*	chrX	22,283,504	22,276,529	
Atp6ap2	NM_001007091	8	7	*Rattus norvegicus*	chrX	22,272,699	22,265,189	
S100a4	NM_012618	0	1	*Rattus norvegicus*	chr2	182,885,080	182,886,301	
S100a4	NM_012618	1	2	*Rattus norvegicus*	chr2	182,886,396	182,887,116	
Sod1	NM_017050	3	2	*Rattus norvegicus*	chr11	29,812,746	29,811,996	
Sod1	NM_017050	2	1	*Rattus norvegicus*	chr11	29,814,314	29,812,763	
Sod2	NM_017051	4	3	*Rattus norvegicus*	chr1	41,865,039	41,864,427	
Sod2	NM_017051	3	2	*Rattus norvegicus*	chr1	41,868,148	41,865,155	
Sod3	NM_012880	1	0	*Rattus norvegicus*	chr14	63,387,093	63,383,027	
Tgfb1	NM_021578	1	2	*Rattus norvegicus*	chr1	80,898,707	80,900,377	
Tgfb1	NM_021578	5	6	*Rattus norvegicus*	chr1	80,910,208	80,910,748	
Timp1	NM_053819	2	1	*Rattus norvegicus*	chrX	12,546,228	12,544,383	
Timp1	NM_053819	5	4	*Rattus norvegicus*	chrX	12,543,722	12,542,718	
Tnf	NM_012675	0	1	*Rattus norvegicus*	chr20	3,661,297	3,661,856	
Tnf	NM_012675	3	3	*Rattus norvegicus*	chr20	3,662,950	3,663,001	
Vim	NM_031140	7	8	*Rattus norvegicus*	chr17	87,854,627	87,855,433	
Vim	NM_031140	4	5	*Rattus norvegicus*	chr17	87,852,234	87,853,124	
Gapdh	NM_017008	2	1	*Rattus norvegicus*	chr4	161,285,787	161,283,854	
Pgk1	NM_053291	2	3	*Rattus norvegicus*	chrX	94,331,155	94,331,330	
Pgk1	NM_053291	7	8	*Rattus norvegicus*	chrX	94,336,859	94,338,987	
HPRT1	NM_012583	7	8	*Rattus norvegicus*	chrX	139,960,354	139,961,091	

### 2.9 Statistical analysis

All data are presented as mean ± SE. Statistical analyses were performed by two‐way ANOVA followed by Tukey's post hoc analysis using GraphPad Prism 8.0 (San Diego, CA). Glomerulosclerosis scoring was analyzed by the Mann–Whitney test. Means were considered significantly different if *p* < .05.

## 3 RESULTS

### 3.1 Systolic blood pressure does not differ between sexes in Dahl S rats

Although studies in other rat models of hypertension exhibit significant BP differences between sexes (Hinojosa‐Laborde et al., 2000), systolic BP was similar (*p* > .05) between sexes in the Dahl SS/Jr rat at 3 and 6 months of age (Table 3).

**Table 3:** Systolic BP does not differ significantly between sexes or over time (*p*, sex = 0.9, *p*, age = 0.06, *p*, interaction = 0.4) *n* = 6‐12/group

Systolic BP mmHg	3 months	6 months
Male	159 ± 9	180 ± 4
Female	165 ± 3	173 ± 12

### 3.2 Female Dahl S rats exhibit maintenance of renal function with attenuated age‐related progression of renal injury compared to males

**Figure 1:**
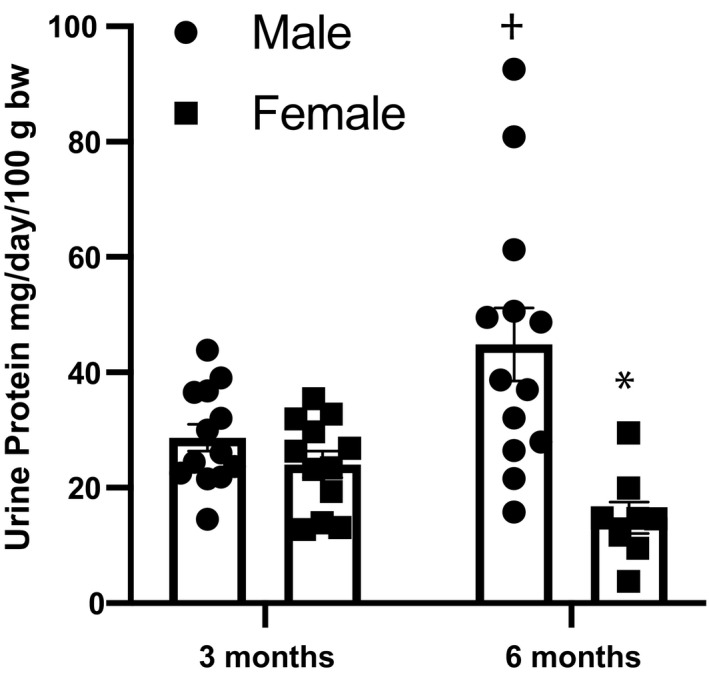
Twenty‐four‐hour protein excretion increases significantly in male Dahl S rats from 3 to 6 months of age but does not in age‐matched female offspring. Proteinuria is also significantly lower in female Dahl S rats at 6 months of age as compared to age‐matched males. n/group: 3 mo male = 13; 3 months female = 12; 6 months male = 13; 6 months female = 8, *p < .05 versus same age male, †*p* < .05 versus same sex at 3 mo. Analysis performed using two‐way ANOVA followed by Tukey's post hoc test

**Figure 2:**
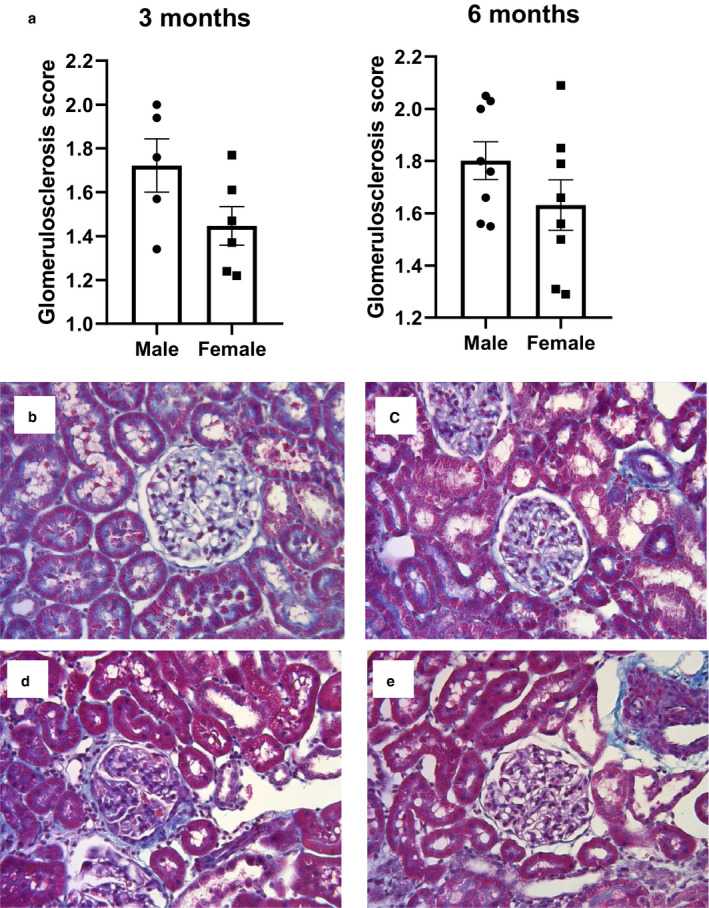
Histological glomerular injury is not different between sexes. (a) Quantification by glomerulosclerosis scoring. (b–e), representative images. (b) Three‐month male, *n* = 5. (c) Three‐month female, *n* = 6. (d) Six‐month male, *n* = 8. (e) Six‐month female, *n* = 8. Analysis performed using Mann–Whitney test

**Figure 3:**
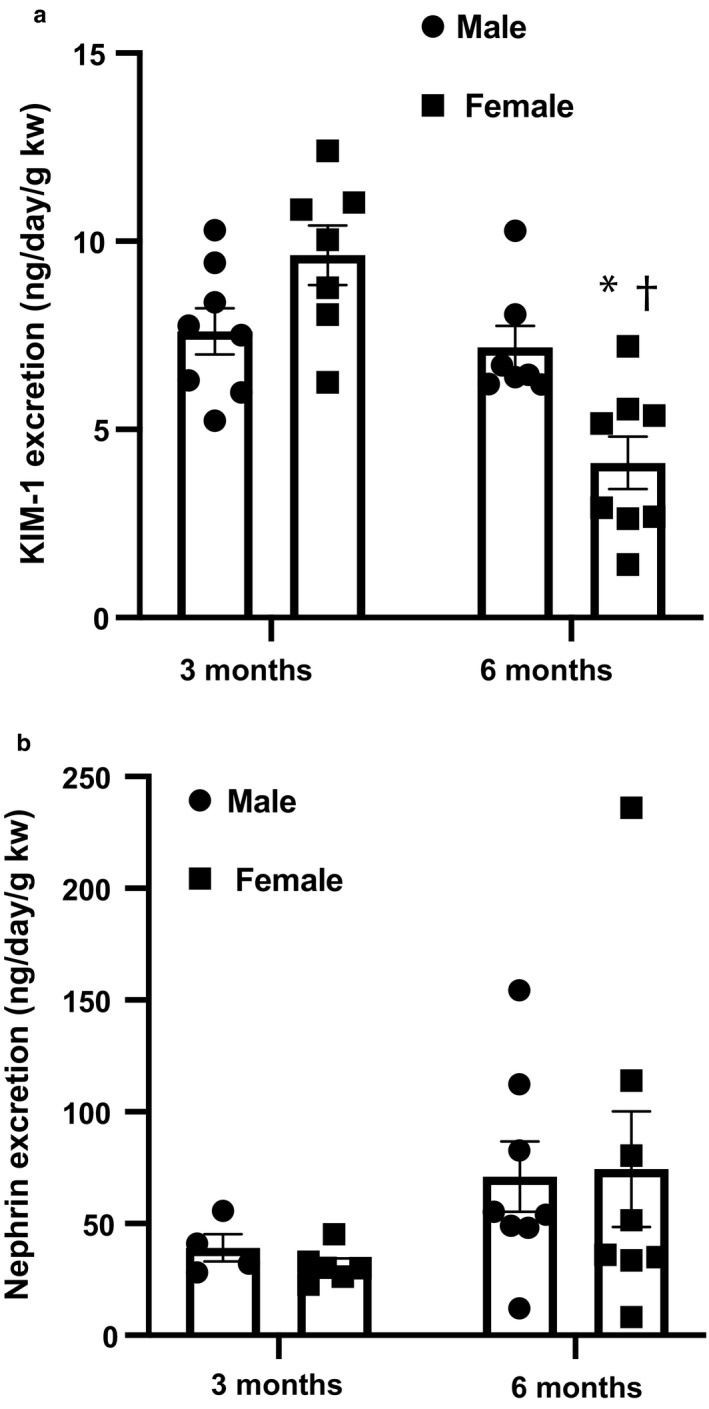
Age‐related advancement of renal injury is moderately attenuated in female offspring. KIM‐1 excretion is significantly lower than that of age‐matched males at 6 months of age, indicating attenuated advancement of renal injury with age. Nephrin excretion is no different between sexes at 3 or 6 months of age. (a) Kidney injury marker‐1 (KIM‐1) measured in urine, n/group: 3 mo male = 8; 3 mo female = 7; 6 mo male = 7; 6 mo female = 8. (*p < .05 versus. same age male, **†** *p* < .05 versus. same sex at 3 mo). (b) Nephrin measured in urine, n/group: 3 months male = 4; 3 mo female = 6; 6 months male = 8; 6 months female = 8. Analysis performed using two‐way ANOVA followed by Tukey's post hoc test

**Figure 4:**
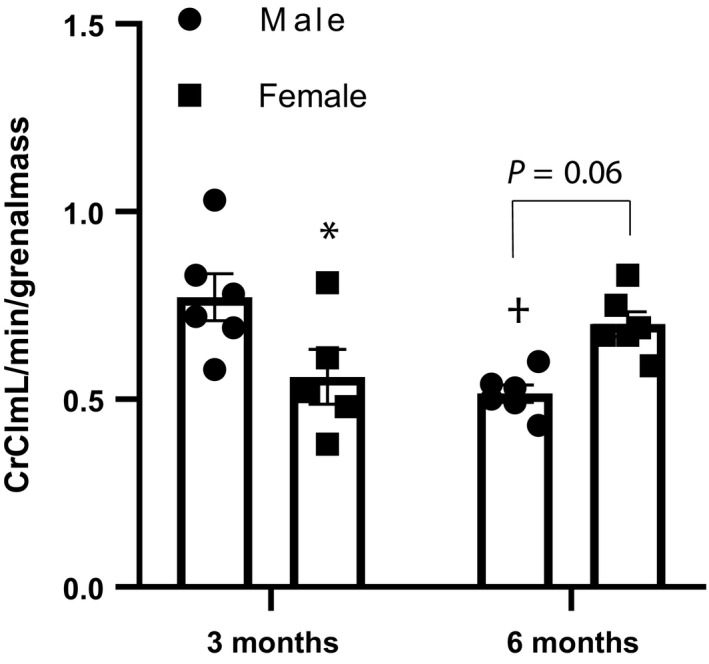
Creatinine clearance is significantly lower in female Dahl S rats at 3 months of age, but is not statistically different at 6 months of age as compared to three months of age (*p* = .2), whereas that of male Dahl S rats decreases significantly (*p* = .01), n/group: 3 months male = 8; 3 months female = 5; 6 months male = 13; 6 months female = 6. (*p*, interaction age x sex < 0.0001, *p < .05 vs. same age male, †*p* < .0001 vs. same sex at 3 months). Analysis performed using two‐way ANOVA followed by Tukey's post hoc test

Although no significant difference exists in systolic BP, females exhibited significantly lower urinary protein excretion at 6 months of age compared to males (Figure 1), indicating less damage to the glomerular filtration barrier, as well as less glomerulosclerosis (Figure 2). This degree of injury was further investigated by using nephrin as a marker of glomerular injury and kidney injury marker‐1 (KIM‐1) as a marker of tubular injury. While no significant sex differences were found in excretion of nephrin at either time point, KIM‐1 excretion was significantly lower in females as compared to males at 6 months of age, in congruency with the proteinuria data (Figure 3). Females also demonstrated no change in creatinine clearance over time, showing no significant difference from 3 to 6 months of age (*p* = .2), while males showed a significant decline over this time period (*p* < .0001). At 3 months, creatinine clearance of females was significantly lower than that of males; however, at 6 months, females were significantly higher due to the lack of change with time compared to the decline seen in male rats (Figure 4).

### 3.3 Female Dahl S rats exhibit significantly higher EC SOD mRNA but no difference in protein expression of SOD isoforms or TBARS excretion

**Figure 5:**
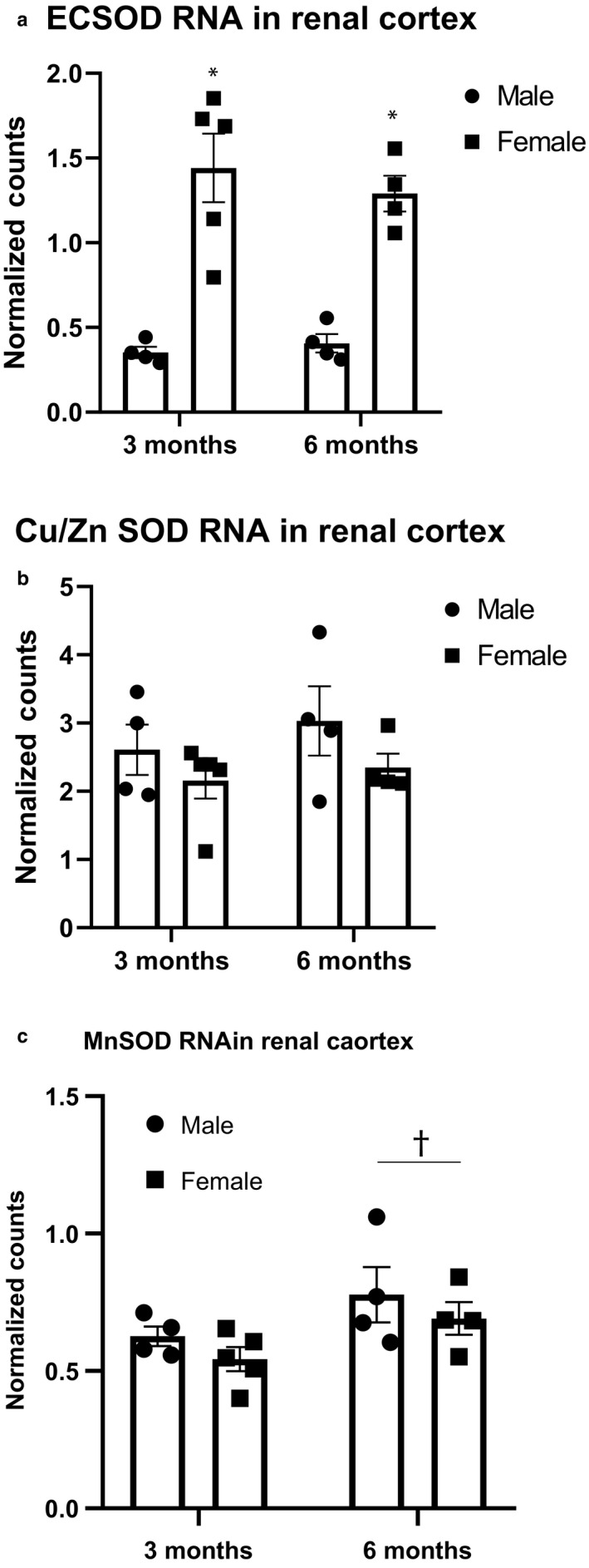
RNA expression of superoxide dismutase isoforms in the renal cortex. Expression levels of Cu/Zn SOD and Mn SOD remain relatively stable in both sexes at both time points, with expression of Mn SOD increasing significantly with age. However, RNA expression of EC SOD in the renal cortex is significantly higher in female Dahl S rats at both 3 and 6 months of age, n/group: 3 months male = 4; 3 months female = 5; 6 months male = 4; 6 months female = 4. (a) SOD3, EC SOD, extracellular isoform, (*p*, sex < .0001, *p < . 5 vs. same age male). (b) SOD1, Cu/Zn SOD, cytoplasmic isoform. (c) SOD2, Mn SOD, mitochondrial isoform, (†*p*, age = .03). Analysis performed using two‐way ANOVA followed by Tukey's post hoc test

**Figure 6:**
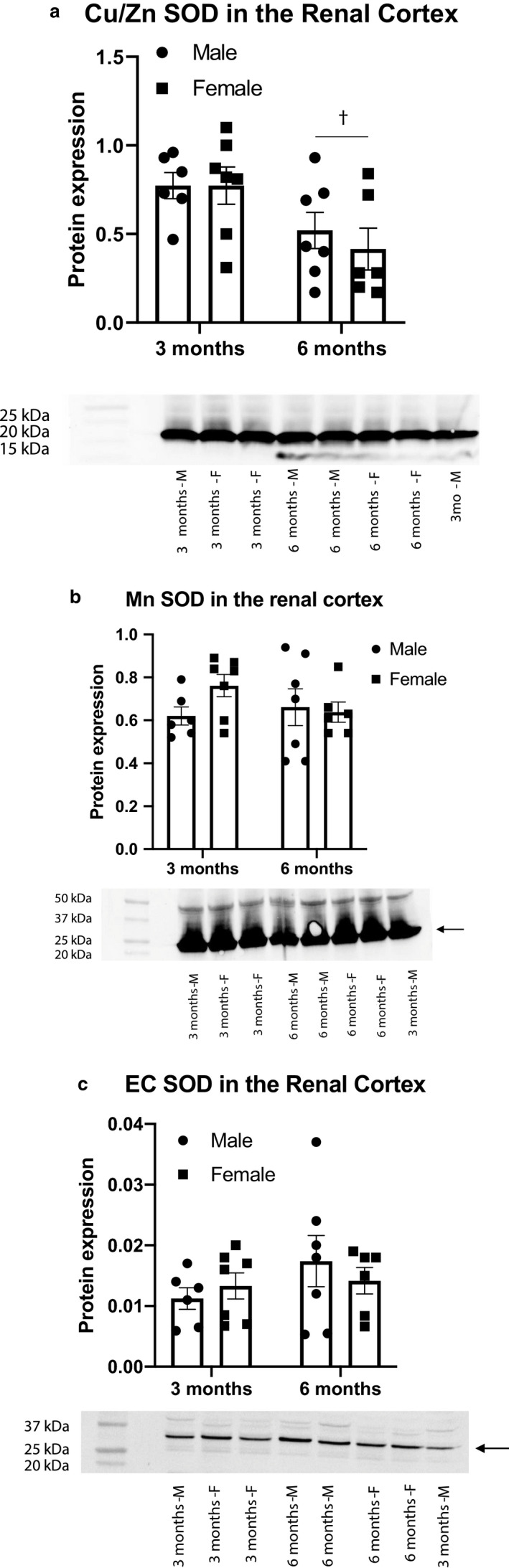
Protein expression of superoxide dismutase isoforms in the renal cortex. Protein loading was controlled by correcting for total protein present in each sample as measured by stain‐free gel imaging, n/group: 3 months male = 6; 3 months female = 7; 6 months male = 7; 6 months female = 6. (a) SOD1, Cu/Zn SOD, cytoplasmic isoform, 16kDa, (†*p*, age = 0.007). (b) SOD2, Mn SOD, mitochondrial isoform, 27kDa. (c) SOD3, EC SOD, extracellular isoform, 130 kDa. Analysis performed using two‐way ANOVA followed by Tukey's post hoc test

**Figure 7:**
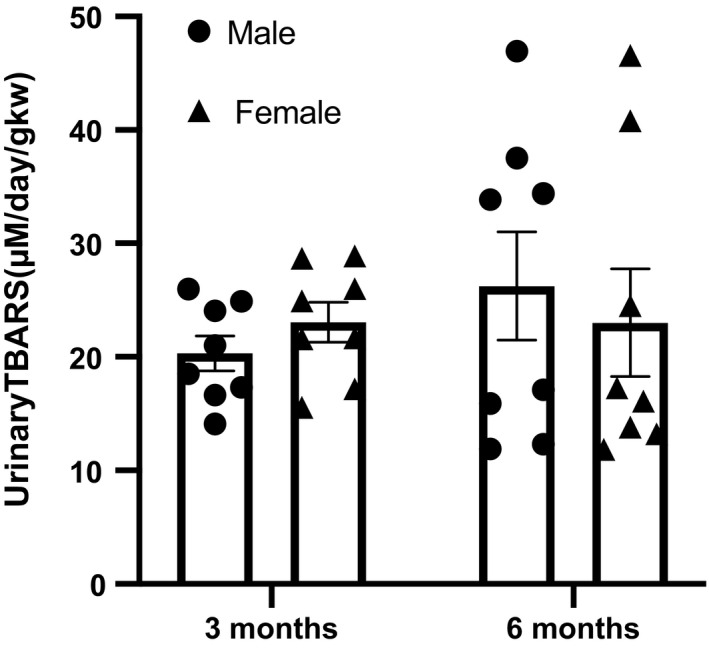
Urinary TBARS does not differ between sexes at either 3 or 6 months of age, *n* = 8 for all groups. Analysis performed by two‐way ANOVA followed by Tukey's post hoc test

We employed targeted RNA sequencing to explore potential mechanisms for the exacerbated renal injury observed in male Dahl S rats (Table 4). While no sex differences were apparent in most genes, this analysis revealed a significant upregulation of EC SOD (SOD3), the extracellular form of the antioxidant enzyme superoxide dismutase, in female Dahl S rats as compared to males (Figure 5a). However, it seems that this difference is isolated to a single isoform, as no differences exist in transcription of Cu/Zn SOD, the cytoplasmic form of the enzyme, or Mn SOD, the mitochondrial form, at either time point (Figure 5b‐c). To further explore the potential role of SOD in the observed renoprotection, we sought to confirm differences at the protein level and determine if there were measurable differences in oxidative stress markers. The difference in transcription levels of EC SOD are not supported by protein expression as determined by western blot, which shows equivocal levels of all isoforms between sexes at each time point (Figure 6a‐c). Furthermore, no sex differences were seen in urinary TBARS at either 3 or 6 months of age (Figure 7).

**Table 4:** Normalized counts of all genes studied via targeted RNA sequencing. (Und = undetectable, *p < .05 vs. age‐matched male)

Gene	3 mo‐Male	3 mo‐Female	6 mo‐Male	6 mo‐Female	*p*, age	*p*, sex	interaction
Agtr1a	0.015	0.020	0.016	0.023	.7	.2	0.9
cat	1.041	1.039	1.723	0.774	.4	.06	0.06
col3a1	0.104	0.087	0.121	0.051	.6	**.04**	0.2
edn1	0.007	0.005	0.012	0.003	.6	.07	0.3
ednra	0.001	0.002	0.001	0.001	.5	.7	0.7
ednrb	0.059	0.079	0.047	0.064	.08	**.02**	0.9
gpx2	0.009	0.018	0.025	0.012	.2	.6	**0.02**
gss	0.595	0.821	0.605	0.605	.4	.3	0.3
hmox1	0.007	0.006	0.005	0.005	.2	.8	1.0
hmox2	Und	Und	Und	Und	—	—	—
hif3a	0.000	0.001	0.003	0.001	.2	.5	0.3
IL10	Und	Und	Und	Und	—	—	—
il17a	Und	Und	Und	Und	—	—	—
IL6	Und	Und	Und	Und	—	—	—
havcr1	0.023	0.048	0.057	0.024	.8	.8	0.08
nox4	0.151	0.097	0.209	0.132	.4	.2	0.8
nphs1	0.027	0.035	0.011	0.023	.2	.5	0.3
lcn2	0.009	0.020	0.017	0.016	.7	.5	0.3
NOs2	Und	Und	Und	Und	—	—	—
NOS3	0.005	0.007	0.003	0.005	.2	.2	1.0
pde5a	0.005	0.022	0.013	0.012	.8	.08	0.06
nphs2	0.103	0.097	0.073	0.079	.5	.7	0.5
prkg2	Und	Und	Und	Und	—	—	—
atp6ap2	0.113	0.162	0.167	0.178	**.04**	.07	0.2
s100a4	0.031	0.028	0.040	0.046	.2	.9	0.7
sod1	2.609	2.156	3.031	2.348	.4	.1	0.7
sod2	0.627	0.542	0.778	0.691	**.03**	.2	1.0
sod3	0.353	1.369*	0.407	1.290*	.7	**<.0001**	0.5
tgfb1	0.019	0.023	0.021	0.018	.7	.9	0.5
timp1	0.033	0.042	0.036	0.031	.6	.8	0.3
tnf	Und	Und	Und	Und	—	—	—
vim	0.141	0.173	0.144	0.124	.4	0.8	0.3

Bold indicates *p* < .05.

### 3.4 Female Dahl S rats maintain NO production over time while males do not

**Figure 8:**
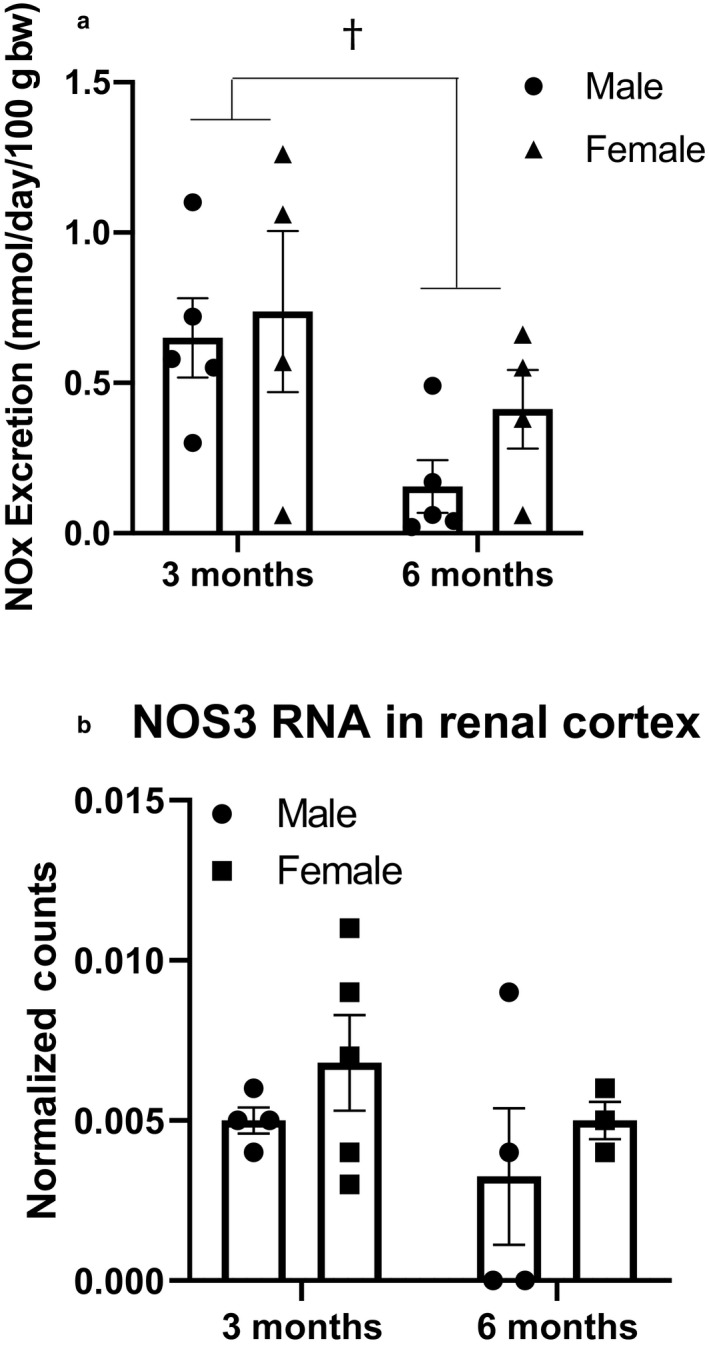
Renal NO generation is reduced in male and female rats with age (*p* = .02), despite a lack of difference in NOS3 transcription or protein expression in the renal cortex. However, there are no significant sex differences at either time point. *n* = 6–8/group. RNA and protein expression of NOS3 do not change over this time and do not differ between sexes. (A) NOx measured in urine, n/group: 3 months male = 5; 3 months female = 4; 6 months male = 5; 6 months female = 4. (b) RNA expression of NOS3 in the renal cortex, n/group: 3 months male = 4; 3 months female = 5; 6 months male = 4; 6 months female = 3. Analysis performed using two‐way ANOVA followed by Tukey's post hoc test

The balance between ROS and NO has been cited as a key to renoprotection, since NO acts to attenuate the harmful effects of superoxide (Ratliff, Abdulmahdi, Pawar, & Wolin, 2016). Urinary excretion of NO metabolites (nitrate/nitrite, NOx) declines over time (*p* = .02); however, no sex differences are present (Figure 8a). No differences exist between sexes or ages in RNA (Figure 8b and Table 4).

## 4 DISCUSSION

The salient findings from this study show that there is a disparate degree of renal injury between male and female Dahl S rats despite similar systolic BP between sexes. We have shown attenuated renal injury and maintenance of renal function in female offspring supported by lower proteinuria and KIM‐1 excretion, in addition to a lack of decline in creatinine clearance by 6 months of age, as compared to age‐matched male rats. Increased urinary excretion of nephrin in male and female rats by 6 months of age confirms the progression of renal injury in this model. While we observed significantly higher mRNA levels of EC SOD in renal cortices of female rats, no difference in protein expression or oxidative stress/antioxidants was apparent. NOS3 expression and NOx excretion were similar between sexes, indicating that the NO pathway likely does not contribute to the exacerbation of renal injury and reduction in renal function with age in male Dahl SS/Jr rats.

One potential mechanism for sex differences in renal injury in Dahl S rats is the differential contribution of ROS to hypertension in male and female animals. Oxidative stress has been shown to directly contribute to hypertension in several rat models, including spontaneously hypertensive rats (SHR), stroke‐prone spontaneously hypertensive rats (SHRSP), DOCA‐salt hypertensive rats, and male Dahl S rats, with many including data that show concurrent reduction of BP when antioxidants are administered (Beswick et al., 2001; Grunfeld et al., 1995; Meng et al., 2003; Schnackenberg et al., 1998; Taylor, Glocka, Liang, & Cowley, 2006; Tian et al., 2007). However, clinical trials of antioxidant use in hypertension have yielded inconsistent results and largely focus on cardiovascular outcomes without reporting outcomes of hypertension‐induced renal disease (Czernichow et al., 2005; Kizhakekuttu & Widlansky, 2010; Ward et al., 2007). Most seem to be limited by the ability to achieve therapeutic levels in vivo or potential side effects, with the most promising being coenzyme Q10 (Kizhakekuttu & Widlansky, 2010).

The limited number of studies on hypertension in female humans and animals suggest that the etiology of disease in this sex may be more complex, possibly due to numerous sex‐specific health events such as pregnancy and menopause (Gillis & Sullivan, 2016; Wenger et al., 2016). Because epidemiologic data show an increase in hypertension in women as they age (Mozaffarian et al., 2016), as well as increased risk for end‐organ damage in premenopausal women (Palatini et al., 2011), it is important to study mechanisms of sex differences independently of BP. For example, Ji et al. (2005) showed a higher degree of renal injury in male rats as compared to female rats with a similar degree of hypertension in a renal wrap model, which may be related to differences in renal NO production between the sexes. Human studies have also demonstrated that ROS are present at higher levels in males as compared to age‐matched females independent of BP (Tomomi et al., 2002).

Salt‐sensitive hypertension in the male Dahl S rat has been shown to cause a sharp decrease in levels of all isoforms of renal medullary SOD and increases in cortical and medullary superoxides compared to those fed a low salt (0.03% NaCl) diet, which are thought to precede elevations in MAP (Meng, Roberts, Cason, Curry, & Manning, 2002). Even those animals on the low‐salt diet showed decreases in renal SOD and increases in renal superoxides compared with Dahl R (salt resistant) rats, suggesting that Dahl S rats on a low‐salt diet exhibit reduction or destruction of SOD, and that further reduction of SOD may be an underlying cause of their salt sensitive hypertension (Meng et al., 2002). However, since these studies did not include female rats, a contribution of this mechanism to sex differences in renal injury and function could not be assessed. Our data support and add to these findings by showing age‐related increases in renal oxidative stress, which may be reflective of a decrease in cortical Cu/Zn SOD expression and increases in nephrin excretion in both sexes.

Bhatia, Elmarakby, El‐Remessy, & Sullivan (2012) showed that male SHR exhibit higher levels of oxidative stress and lower antioxidant potential during Ang II‐induced hypertension, and that the male‐specific increase in BP with Ang II can be attenuated using the antioxidant apocynin. However, the key enzymatic difference in this study was in renal cortical NADPH oxidase, while SOD expression and activity were similar among groups. These data, combined with the present study, suggest that the molecular mechanisms differ among models of hypertension, in addition between sexes within the same model.

Superoxide reacts with NO at a faster rate than it does with SOD (Gryglewski, Palmer, & Moncada, 1986; Omar, Cherry, Mortelliti, Burke‐Wolin, & Wolin, 1991; Rubanyi & Vanhoutte, 1986). A study by Grunfeld et al. (1995) in SHRSP vascular endothelial cells demonstrated that these cells produce more superoxide than those of normotensive controls and concordantly less NO, which is replenished by addition of SOD. This suggests that superoxide reduces NO levels with a contributory effect of reduction in SOD levels. Indeed, another study suggests that the mechanism of oxidative stress‐induced hypertension is via its inactivation of NO (Schnackenberg et al., 1998). The reaction between NO and superoxide also leads to the formation of peroxynitrite, perpetuating the cascade of endothelial dysfunction by damaging endothelial cells and preventing NO‐induced vasorelaxation (Beckman & Koppenol, 1996). The data shown here support the contribution of a harmful NO/ROS balance to age‐ and hypertension‐related renal injury by showing an increase in oxidative stress, reduction in Cu/Zn (cytoplasmic) SOD, and decrease in NO excretion products (NOx) from 3 to 6 months of age.

NOS3 is a major producer of NO not only in the renal vasculature as previously thought (Ujiie, Yuen, Hogarth, Danziger, & Star, 1994), but also in the tubules (Allcock, Hukkanen, Polak, Pollock, & Pollock, 1999). Upregulation of NOS3 has been shown to play a protective role against hypertensive renal damage in rodent models, including the Dahl S rat (Allcock et al., 1999; Tashiro, Yogo, Serizawa, & Endo, 2015). Furthermore, the combination of drug‐induced maintenance of NOS3 activity and reduction in ROS has been shown to protect against hypertensive end‐organ damage (Zhou et al., 2004). NOS3 protein expression in the renal cortex of Sprague–Dawley rats (*SD*) previously showed sex differences with age, where expression decreased significantly in males but not females (Erdely, Greenfeld, Wagner, & Baylis, 2003). However, we found no difference between sexes in either RNA or protein expression of NOS3, suggesting that differences in renal injury may lie downstream of NOS. The *SD* rat is a progenitor strain for both the Dahl S rat and the Dahl R rat, but the two descendant strains have also previously shown significant differences in NOS3 expression, where Dahl R rats show twice the level of NOS3 mRNA expression of Dahl S rats (Kobayashi et al., 2008). The current study suggests that female Dahl S rats may be better able to maintain functional production of NO, as evidenced by their maintenance of NOx excretion with age. It is also possible that the increased superoxide seen in male rats results in greater inactivation of NO with resultant decreases in NO bioavailability.

The current study exhibits an increase in transcription of EC SOD in the healthier female rats with no detectable difference in protein expression. True elevation of EC SOD resulting in increased protein expression and activity would be consistent with a response to renal damage induced by infiltrating immune cells. Upregulation of EC SOD has been shown to reduce tubulointerstitial fibrosis, as well as expression levels of profibrotic factors TGF‐β and collagen I in a model of diabetic nephropathy (Kuo et al., 2015). Reduced levels of EC SOD and increases in oxidative stress have also been shown to play a key role in the progression of proteinuric kidney disease in several animal models, including ADR‐induced nephropathy, Ang II‐induced renal injury, and albumin‐overload proteinuria (Tan et al., 2015). However, the difference we saw in EC SOD RNA did not result in a similar difference in protein expression of EC SOD in the renal cortex. EC SOD undergoes significant posttranslational modification, including C‐terminal processing and N‐glycosylation, which is necessary to its secretion and function (Olsen et al., 2004; Ota, Kizuka, Kitazume, Adachi, & Taniguchi, 2016). One possibility for the results we show is that even though male rats seem to exhibit similar protein expression of EC SOD as females, there could be some processing defect that prevents modification necessary for secretion and effective action of EC SOD in the renal cortex. Conversely, there could also be increased proteolytic cleavage of EC SOD, preventing its secretion and altering tissue distribution of the enzyme.

While the tail cuff method used in these studies provided an opportunity to measure large groups of animals repeatedly and noninvasively, tail cuff readings are likely less sensitive than telemetry and the restraints necessary to tail cuff measurement provide additional stress to the animals not seen in a telemetry setting. There is also a historically consistent difficulty in accurately assessing the NO production and activity, the level of oxidative stress, and the physiologic capability to mediate oxidative stress within the kidney. The short half‐lives of NO and many markers of oxidative stress yield an unavoidable level of inaccuracy in most or all available assays. While enzyme expression levels can yield an idea of the kidney's response to hypertension and renal injury, this does not always correlate with activity levels, and activity levels do not always correlate with functional effects, due to increases in degradation or other physiologic interference. Another limitation is the fact that the 3‐ and 6‐month groups consisted of different animals (i.e., a single animal was not followed through all time points), a limitation necessitated to obtain blood and renal tissue at each time point. However, future studies will aim to track the renal function of individual animals over the course of time. Based on these results, we suggest that a reduced NO: ET‐1 balance in male rats contributes to the observed sex differences in renal injury and function. Further study of these differences in animal models of hypertensive kidney disease is necessary to determine the true mechanism of preserved renal function in female animals.

### 4.1 Perspectives

The Dahl S rat is an established model of salt‐sensitive hypertension and chronic kidney disease (CKD) (Dahl & Schackow, 1964; Tanada et al., 2017), as well as spontaneous, superimposed preeclampsia (Gillis, Williams, Garrett, Mooney, & Sasser, 2015) that develops hypertension with age in the absence of high salt. While short term and chronic effects of hypertension and CKD have been studied in this model, little has been demonstrated regarding the sex differences present in the Dahl S rat. Sex differences are well established in both human patients with hypertension (Wenger et al., 2016) and in several animal models of hypertension (Crofton & Share, 1997; Haywood & Hinojosa‐Laborde, 1997; Reckelhoff, 2001; Rowland & Fregly, 1992; Xue et al., 2005), and are theorized to contribute heavily to the etiologies of various forms of hypertension (Gillis & Sullivan, 2016). Women with chronic hypertension have a odds ratio of developing superimposed preeclampsia of 10.07 as compared to the risk of preeclampsia in their normotensive peers (Bateman et al., 2012). Therefore, elucidation of sex differences in our model of hypertension and CKD will lead to new insights on the etiology of hypertensive renal injury and hopefully to factors that predispose such women to the development of further disease such as preeclampsia. We also observed increasing oxidative stress in both sexes with age; however, clinical trials studying antioxidants in the treatment of hypertension have yielded mixed results (Czernichow et al., 2005; Schneider et al., 2005; Ward et al., 2007), likely due to variability in employed methods, and are not structured to compare results between sexes. Therefore, continued study is required to determine the exact contribution of these pathways to sex differences in hypertension.

## CONFLICT OF INTEREST

The authors have no disclosures to report.

## AUTHOR CONTRIBUTIONS

HRT and JMS conceived and designed research; HRT, ACJ, and ELD performed research experiments; HRT, MRG, and ELD analyzed data; HRT interpreted results of experiments, prepared figures, and drafted manuscript; HRT, ACJ, ELD, MRG, and JMS edited and revised manuscript; HRT, ACJ, ELD, MRG, and JMS approved the final version of the manuscript.
